# Proof-of-Principle of Onchocerciasis Elimination with Ivermectin Treatment in Endemic Foci in Africa: Final Results of a Study in Mali and Senegal

**DOI:** 10.1371/journal.pntd.0001825

**Published:** 2012-09-13

**Authors:** Mamadou O. Traore, Moussa D. Sarr, Alioune Badji, Yiriba Bissan, Lamine Diawara, Konimba Doumbia, Soula F. Goita, Lassana Konate, Kalifa Mounkoro, Amadou F. Seck, Laurent Toe, Seyni Toure, Jan H. F. Remme

**Affiliations:** 1 Direction Nationale de la Santé, Bamako, Mali; 2 Ministere de la Santé et de la Prévention, Dakar, Senegal; 3 Multi-disease Surveillance Centre, Ouagadougou, Burkina Faso; 4 University Cheikh Anta Diop, Dakar, Senegal; 5 Ornex, France; Ghana Health Service, Ghana

## Abstract

**Background:**

Mass treatment with ivermectin controls onchocerciasis as a public health problem, but it was not known if it could also interrupt transmission and eliminate the parasite in endemic foci in Africa where vectors are highly efficient. A longitudinal study was undertaken in three hyperendemic foci in Mali and Senegal with 15 to 17 years of annual or six-monthly ivermectin treatment in order to assess residual levels of infection and transmission, and test whether treatment could be safely stopped. This article reports the results of the final evaluations up to 5 years after the last treatment.

**Methodology/Principal Findings:**

Skin snip surveys were undertaken in 131 villages where 29,753 people were examined and 492,600 blackflies were analyzed for the presence of *Onchocerca volvulus* larva using a specific DNA probe. There was a declining trend in infection and transmission levels after the last treatment. In two sites the prevalence of microfilaria and vector infectivity rate were zero 3 to 4 years after the last treatment. In the third site, where infection levels were comparatively high before stopping treatment, there was also a consistent decline in infection and transmission to very low levels 3 to 5 years after stopping treatment. All infection and transmission indicators were below postulated thresholds for elimination.

**Conclusion/Significance:**

The study has established the proof of principle that onchocerciasis elimination with ivermectin treatment is feasible in at least some endemic foci in Africa. The study results have been instrumental for the current evolution from onchocerciasis control to elimination in Africa.

## Author Summary

The control of onchocerciasis, or river blindness, is based on annual or six-monthly treatment with ivermectin of populations at risk. This has been effective in controlling the disease as a public health problem but it was not known whether it could also eliminate infection and transmission to the extent that treatment could be safely stopped. Many doubted that this was feasible in Africa. A study was undertaken in three hyperendemic onchocerciasis foci with seasonal transmission in Mali and Senegal where treatment has been given for 15 to 17 years. As a result of this treatment, infection and transmission levels had fallen everywhere below postulated thresholds for elimination. Treatment was therefore stopped in each focus. Follow-up evaluations up to 5 years after the last treatment showed no evidence of recrudescence after stopping treatment but instead a consistent decline in infection and transmission levels, reaching zero in two sites. The study has established the proof-of-principle that onchocerciasis elimination with ivermectin treatment is feasible in at least some endemic foci in Africa. The results of the study have greatly contributed to the current evolution from onchocerciasis control to elimination in Africa.

## Introduction

Onchocerciasis control is currently nearly exclusively based on large-scale treatment with ivermectin [1]. Annual or six monthly treatment of all eligible members of high risk communities effectively controls the disease as a public health problem [2]–[4], and following the donation of the drug free of charge for as long as needed by the manufacturer in 1987, large-scale ivermectin treatment programmes have been established in nearly all endemic areas in Africa where over 99% of all cases in the world are found [5]. The African Programme for Onchocerciasis Control (APOC) was established in 1995 to support the establishment of community directed treatment with ivermectin (CDTi) in all areas where onchocerciasis was a public health problem in 19 African countries [6]. The CDTi strategy has been very successful in ensuring sustained high treatment coverage and by the year 2010 some 75 million people at risk were treated annually with ivermectin in the APOC countries [7]. The remaining 11 endemic African countries had been supported since 1975 by the Onchocerciasis Control Programme in West Africa (OCP) [8]. OCP was based on vector control to which ivermectin treatment was added in 1988. Following the successful completion of the vector control activities and the closure of the OCP programme in 2002, large-scale ivermectin treatment using the CDTi strategy has been maintained by these countries themselves.

As a result of these sustained ivermectin treatment activities, nearly all endemic areas in Africa are under annual ivermectin treatment and the control of onchocerciasis as a public health problem has already been achieved in the majority of these areas [9].

Following this success, the principal question became how long these treatments needed to continue and whether in the long term it would ever be possible to eliminate onchocerciasis infection and transmission with ivermectin treatment so that treatment could be safely stopped. Epidemiological models predicted that elimination would be feasible in the long term [10], and in the Americas where most onchocerciasis foci are small and most vectors relatively inefficient, elimination has been set as the objective by the Onchocerciasis Elimination Program for the Americas (OEPA) [11], [12]. However, in the absence of empirical evidence on the feasibility of elimination in Africa, most experts doubted that elimination would be possible in the African continent where onchocerciasis is highly endemic over vast areas, and where the vectors are highly efficient and some vector species can migrate over long distances [13]–[15].

In order to study this question, a longitudinal study was undertaken in three onchocerciasis endemic foci in Mali and Senegal. The three foci were among the first areas where large scale ivermectin treatment was started in Africa and by 2006 they had received 15 to 17 years of ivermectin treatment. Interim epidemiological evaluations had indicated that the prevalence of infection had fallen to very low levels [16]. Because of the duration of treatment and the promising interim evaluation results, it was considered that if elimination with ivermectin treatment would be feasible in endemic foci in Africa, these would be the foci where this could be first demonstrated. The aim of the study was to determine if after 15 to 17 years of ivermectin treatment onchocerciasis infection and transmission levels had fallen so low that transmission would be unlikely to sustain itself, and then to test this hypothesis by actually stopping treatment and undertaking follow-up surveys for another 3 years to confirm that was no recrudescence in infection and transmission after cessation of treatment.

The study began in 2006 and was completed in 2011. The first results of the study covering the period 2006 to mid 2008 have been reported by Diawara et al [17]. The final results of the study, including the full results for the 3-year follow-up evaluations after cessation of treatment, are reported here.

## Methods

### Study sites

**Figure 1:**
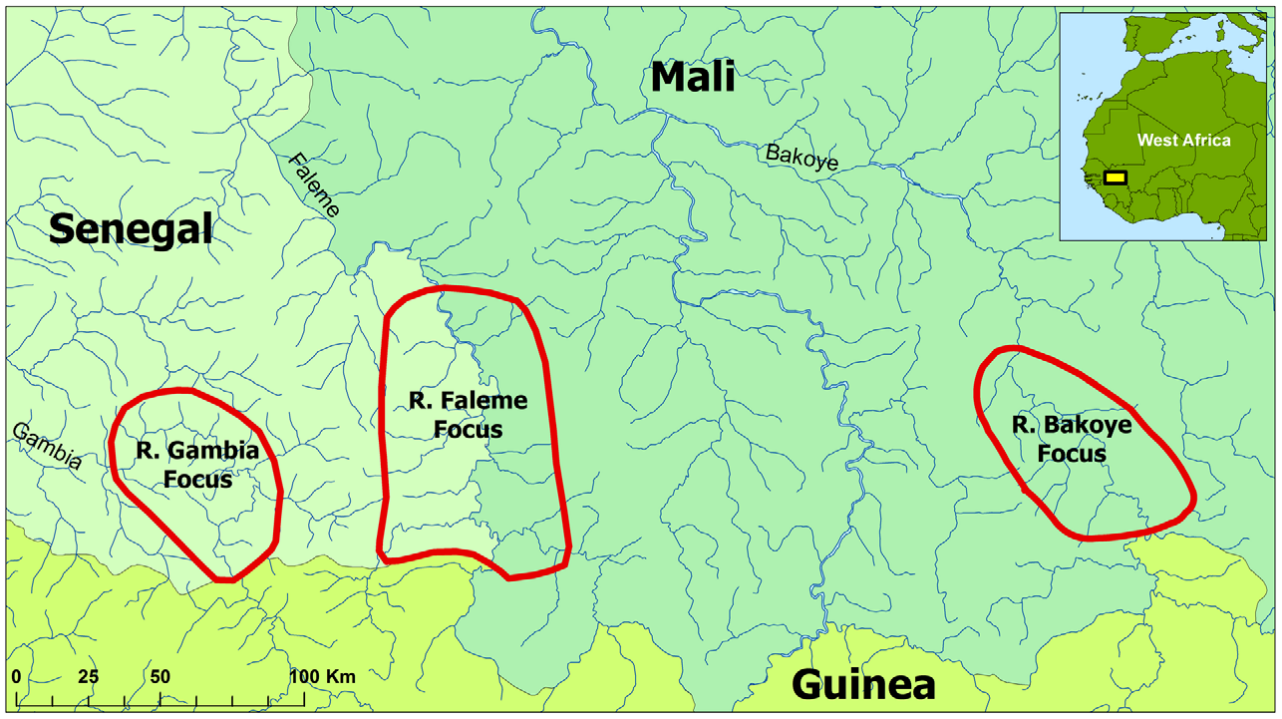
Location of the three study foci in Mali and Senegal.

The study was undertaken in three onchocerciasis foci along the River Bakoye in Mali, the River Gambia in Senegal, and the River Faleme on the border of the two countries (figure 1). These three areas were part of the Western Extension area of the OCP where onchocerciasis control has been exclusively based on ivermectin treatment which started in 1988–1989. According to skin snip surveys undertaken by the OCP before the start of control, in each of these three foci there were hyperendemic villages, i.e. villages with a prevalence of microfilaridermia ≥60% or a Community Microfilarial Load (Cmfl, the geometric mean number of microfilariae per skin snip among adults aged 20 years and above) >10 microfilariae per skin snip (mf/s) [18]–[20]. In the River Gambia focus, 8 out of 22 surveyed villages had a Cmfl >10 mf/s (range 12.0 to 48.1 mf/s) [21]. In the River Bakoye focus 5 out of 11 surveyed villages had a Cmfl >10 mf/s (range 10.2 to 21.6 mf/s) and in the River Faleme focus this was the case for 3 out of 27 surveyed villages (range 13.3 to 21.0 mf/s) [22]. The rural population of the three foci has about the same size with 20,000 to 30,000 people living in 75 to 94 villages per site. In the R. Gambia focus there is also one town with a population of about 18,000.

The onchocerciasis vectors in the study areas are the savanna vectors *Simulium sirbanum* and *Simulium damnosum* s.s. and transmission is limited to the rainy season when the rivers flow from about July to December. All three areas are isolated with respect to long-distance migration of the *Simulium* vectors except for the first few weeks of the rainy season. During the dry season, the rivers do not flow and there are no blackflies. At the beginning of the rainy season, when the Inter-tropical-conversion-zone (ITCZ) moves to the north, the breeding sites are reinvaded by simuliids from the south (mainly *S. sirbanum*) that migrate with the prevailing winds and start the repopulation of the breeding sites [15], [23], [24]. After a few weeks, when the winds change, this long distance migration stops and the vector population becomes purely local with virtually no migration from outside or from neighboring river basins. All river basins involved in this migration pattern are either free from onchocerciasis or under large-scale ivermectin treatment since 1990. For the R. Bakoye, *S. dieguerense* has also been reported but this is a non-migratory *Simulium* species that only plays a local role in onchocerciasis transmission [25].

Along all three rivers there are onchocerciasis endemic villages downstream of the study areas but their endemicity levels are generally lower and they are all covered by the same national ivermectin treatment programs of Mali and Senegal. The neighboring river basins are also endemic for onchocerciasis and undergoing ivermectin treatment. Hence, the three study areas cannot be considered completely isolated areas, but rather as the most endemic sections of onchocerciasis zones along three rivers that are fully covered by the national ivermectin treatment programs.

Ivermectin treatment was given annually in the R. Bakoye and R. Faleme, and at six monthly intervals in the R. Gambia making this the only onchocerciasis endemic area in Africa where six monthly treatment with ivermectin has been given for more than 10 years. The months of treatment were April or May, just before the rainy season, in order to optimize the impact of treatment on transmission. In the R. Gambia there was a second round of treatment in October or November of each year. In the R. Gambia the first round of treatment was given in 1988 and in the other two foci in 1989. The treatment programs were introduced stepwise, covering only the most endemic villages during the first year and gradually expanding the coverage to all villages over the next few years. Hence the number of years that each village has received treatment by the time of the study ranged from 14 to 19 years. We have defined the number of years of ivermectin treatment for each study area as the number of years that all first line villages had been under ivermectin treatment [17]. For the R. Gambia this was 17 years, for the R. Faleme 16 years and for the R. Bakoye 15 years.

During the first few years (1988 to 1991) treatment coverage was not yet satisfactory at 59% to 69% of the total population, but from 1992 onwards the reported treatment coverage was high at 75% to 89% of the total population (corresponding to some 89% to 100% of eligibles). The only exception was the year 1997 when there was a temporary drop in coverage following an abrupt change in drug delivery policy.

More detailed information on the study sites and ivermectin treatment history is provided in the first article on the study [17]

### Study design

Onchocerciasis elimination is defined as the reduction of local onchocerciasis infection and transmission to such low levels that transmission can no longer sustain itself and treatment can be safely stopped without risk of recrudescence of infection and transmission [26].

**Figure 2:**
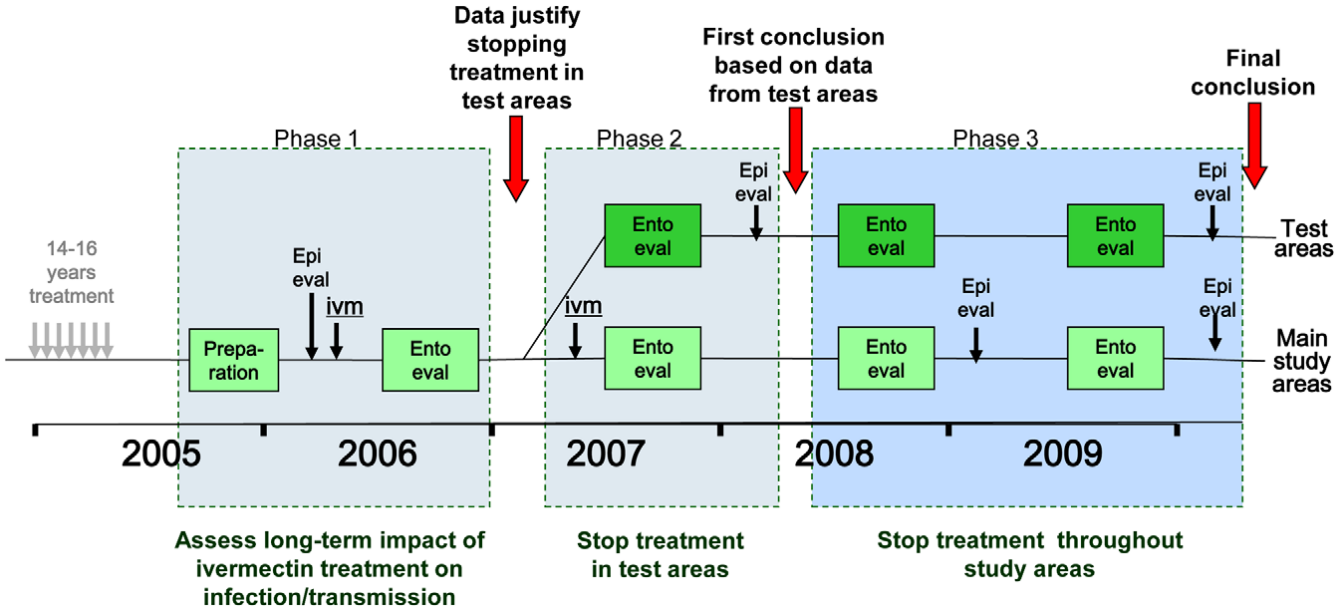
Study design.

To assess whether elimination has been achieved in the three study areas, the study was designed in three phases (figure 2). The aim of the first phase was to undertake a detailed assessment of onchocerciasis infection and transmission levels after 14 to 17 years of treatment. Skin snip surveys were to be undertaken in a stratified random sample of some 40 villages in each study site, and transmission monitored for a full transmission season through entomological evaluations in 4 to 6 fly-catching points per study site. If the observed infection and transmission levels in a study site were below predefined thresholds (see section on indicators below), phase 2 would start in which treatment would be stopped in a test area of 5–8 villages located around one of the catching points in the study site. The effect of stopping treatment on infection and transmission would be evaluated by epidemiological surveys 20 to 22 months after the last treatment in the test villages, and by entomological evaluation in all catching points during another full transmission season. If there was no recrudescence of infection and transmission in the test area, phase 3 would start in which treatment would be stopped throughout the study site and infection and transmission monitored for another two years in all sample villages and catching points. Detailed information on the dates when the different treatment and evaluation activities were undertaken in each of the study foci is provided in the results section.

**Figure 3:**
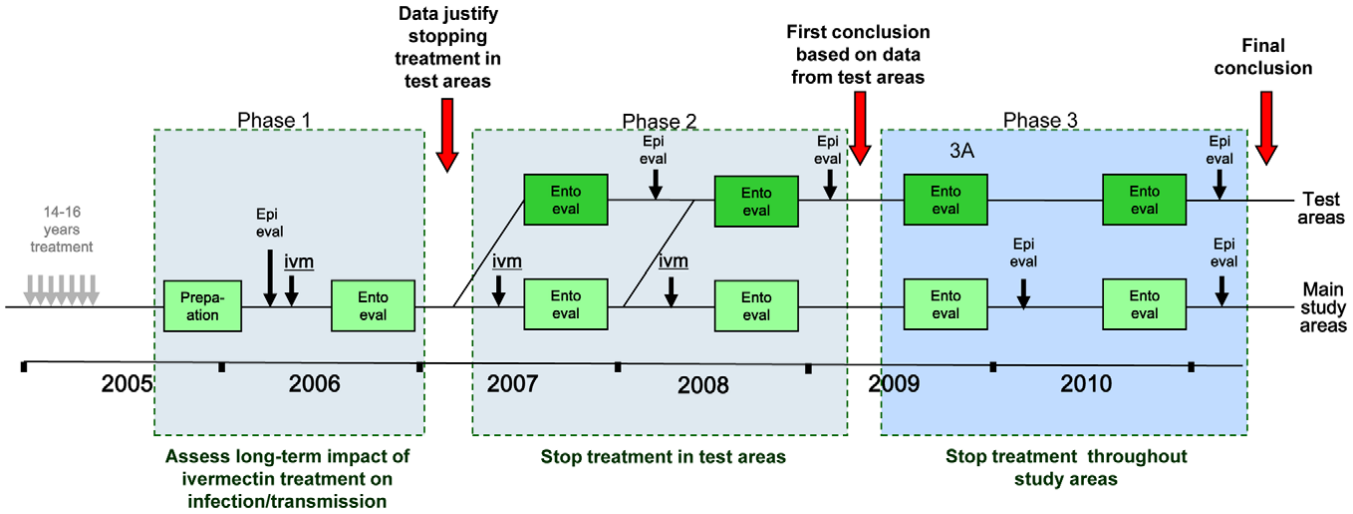
Modified study design for the R. Faleme focus.

In the R. Bakoye and the R. Gambia foci, the study has followed the original design. In the R. Faleme focus one step was added to phase 2 to collect information from two additional test areas in the southern part of the focus, where the phase 1 results were less clear, before making the decision to proceed to phase 3 and stop treatment throughout the focus. Hence, in the R. Faleme site the study has lasted one year longer than in the other two sites (figure 3).

### Epidemiological evaluation methods

Skin snip surveys were done in all selected evaluation villages. In each village, all persons above the age of 1 year who agreed to participate (or whose parent agreed for them to participate in the case of children) were examined for onchocerciasis infection. The surveys used established skin snip examination methods in which the national onchocerciasis teams have been trained by the OCP. Two skin snips were taken from the iliac crests with a 2 mm Holth corneoscleral punch and microscopically examined after incubation for 30 minutes in distilled water (and a further 24 hours in saline for negative skin snips) for the presence and number of *O. volvulus* microfilariae [27]. The numbers of microfilariae were counted and the results recorded for each person examined. Basic information on the migration history for each person during the last 10 years before the survey was also collected. The prevalence of mf was estimated as the percentage of examined persons who had microfilariae in at least one skin snip. Confidence intervals for the prevalence were calculated using the Clopper-Pearson method.

### Entomological evaluation of onchocerciasis transmission

**Figure 4:**
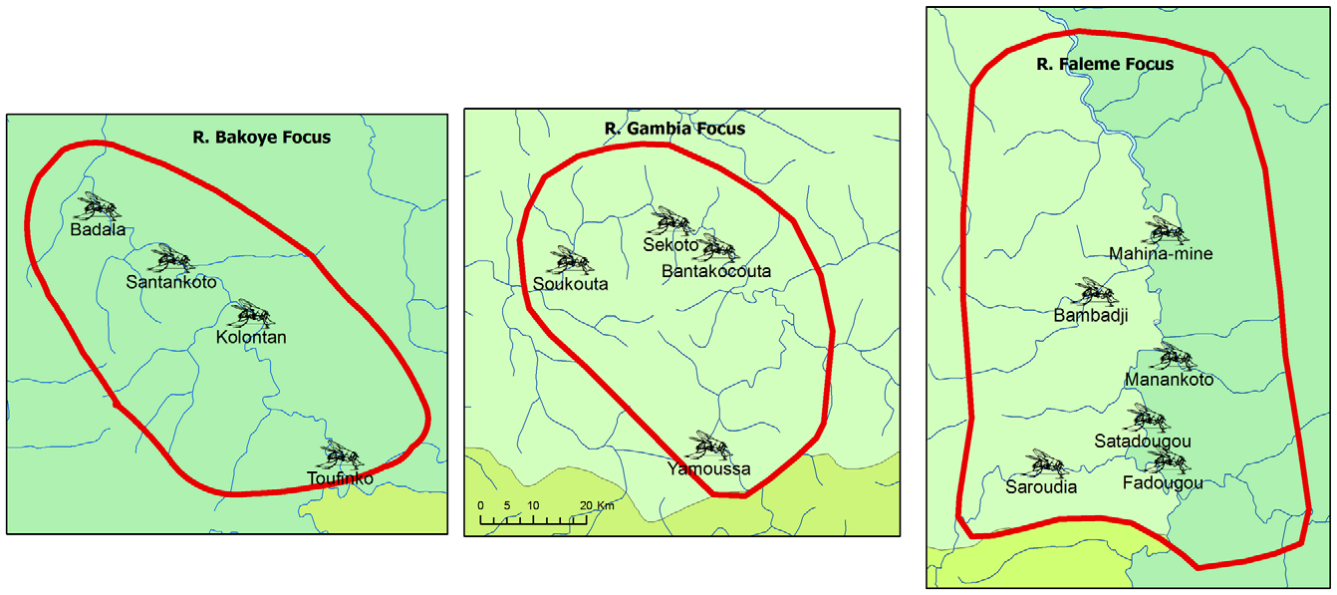
Location of the fly catching points in the three study sites.

During each year, a detailed entomological evaluation was done throughout the transmission season in order to determine the levels of *O. volvulus* transmission. Four vector catching points were selected for the R. Bakoye and R. Gambia and six for the R. Faleme which covers a larger area in two countries. The location of the catching points is shown in figure 4. Every week, 3 days of capture were carried out at each catching point during the transmission period which generally covers 5 to 6 months per year (July/August to November/December). Flies were collected using the method of bulk catches with a team of 3 to 4 fly catchers working from 7 AM to 6 PM. Each daily catch was preserved in 80% alcohol and sent to the DNA laboratory of the Multi-Disease Surveillance Centre (MDSC) in Ouagadougou, Burkina Faso [28]. In the laboratory, the flies were rinsed with distilled water, the heads separated from the bodies and sorted in lots for DNA extraction. The purified DNA was used as a substrate in an O-150 (an *Onchocerca*-specific DNA sequence) PCR, and the resulting product classified by hybridization to the *O. volvulus*-specific oligonucleotide probe OVS-2 [29]. A computer program (Poolscreen) was used to translate the molecular biology data obtained from screening pools of 300 flies into an estimate of the infectivity rate in the vector population [30].

### Indicators

The two main indicators of onchocerciasis infection and transmission used in the present study are the prevalence of microfilariae in the skin in the human population and the vector infectivity rate as measured by the number of flies with *O. volvulus* L3 (infective) larvae in the head per 1,000 flies (FLH/1,000). Based on model predictions as well as large-scale experience in the OCP, provisional thresholds have been defined for these indicators below which the remaining infection and transmission levels are so low that they would die out naturally, even in the absence of any intervention, and when treatment can therefore be safely stopped without risk of recrudescence [17]. The thresholds for the prevalence of infection were defined as a microfilarial prevalence <1% in 90% of sample villages, and a prevalence <5% in 100% of sample villages. The threshold for vector infectivity was defined as 0.5 FLH per 1,000 flies. To ensure that a sample with 0 FLH would imply that the infectivity rate was with 95% confidence below the threshold of 0.5 FLH per 1000 flies, a minimum of 3900 flies was to be analyzed per catching point [30].

The above thresholds were provisional thresholds to guide decision making and analysis in the current study. One of the objectives of the study was to review these thresholds, and revise them as required, based on the final study results.

### Research ethics

Ethical review and clearance of the research protocol, research instruments and informed consent procedures were obtained from the national ethical review boards of the ministries of health in Mali and Senegal, and continuing ethical review was ensured by the ethical review committee of the World Health Organization. Community meetings were held in all villages to explain the research objectives and procedures, and the right of each individual to decide whether to participate in the examinations or not. Before each examination, each individual who had voluntarily come to the examination point and agreed to participate signed, or put a thumb print if not literate, on the examination form to indicate consent. For children, one of the parents or the responsible guardian would sign the examination form. The use of community meetings to discuss the research project and the right of individuals to refuse participation in the examination was considered the most culturally appropriate and effective method for providing the necessary information to community members, and this approach was approved by both the national ethical review boards and the WHO ethical review committee. Community leaders approved the use of the selected locations on the river banks as vector catching points.

## Results

The results for phase 1 and phase 2 of the study have been reported previously by Diawara et al [17]. In this article we report the results of the final evaluations during phase 3 after cessation of ivermectin treatment throughout the study areas.

### R. Bakoye focus, Mali, annual treatment

In the R. Bakoye focus, the last round of ivermectin treatment was given in May 2006 in the villages in the test area, and in May 2007 in all other villages in the focus.

During phase 3, skin snips surveys were done in the same 40 villages that had been surveyed during phase 1 before the cessation of treatment. Of these 40 villages, 20 were surveyed in February 2009 during phase 3A, 21 months after the last treatment. Another 20 villages were surveyed in May 2010 during phase 3B, 36 months after the last treatment in 15 villages from the main area and 48 months after the last treatment in 5 villages that were located in the test area where treatment was stopped one year earlier.

**Figure 5:**
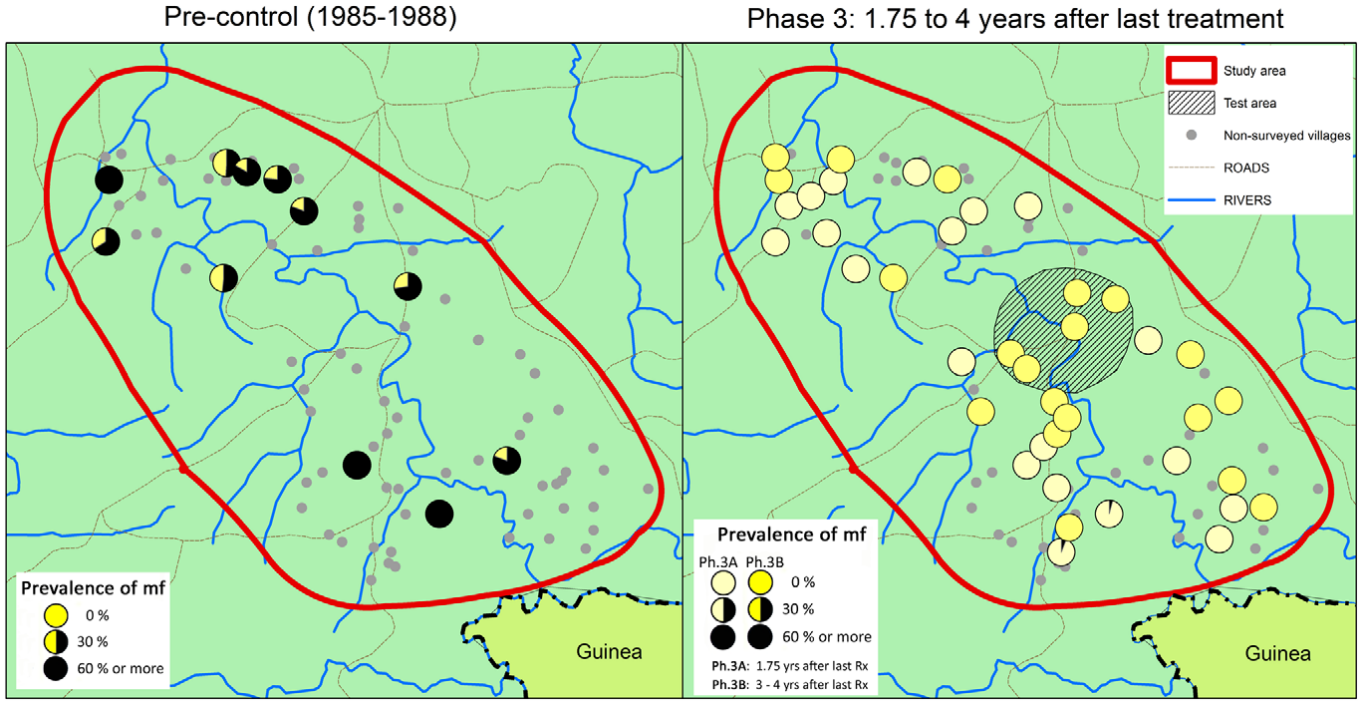
Prevalence of onchocerciasis infection in the R. Bakoye focus before the start of treatment and 1.75–4 years after the last treatment round.

The results of the epidemiological surveys are shown in figure 5 and table 1. As reported previously, the overall prevalence of mf had fallen from a precontrol prevalence of 43.4% to 0.26% after 14 years of treatment. The phase 3 results show that after cessation of treatment, the prevalence of mf continued to decline. In phase 3A, only 2 mf positives (0.05%) were detected out of 3739 persons examined. It concerned one male of 31 years who had a relatively high mf density with 34 and 81 mf in the skin snips from the left and right iliac crests respectively, and who reported to have been treated only twice, once in 1990 and once in 2000. The other was a male of 55 years with 0 and 10 mf in the two skin snips who reported to have been treated only three times in 1994, 1997 and 2007. These two individuals had not been examined during the phase 1 surveys. In phase 3B no mf positives were detected among the 3520 persons examined 3 to 4 years after the last treatment.

**Table 1:** Trend in prevalence of mf in the R. Bakoye focus.

	Pre-control (1988–1990)*	Phase 1* (after 14 years Rx)	Phase 3a (1.75 yrs after last Rx)	Phase 3b (3–4 yrs after last Rx)
Villages surveyed	11	40	20	20
Census population	2,421	9,868	5,816	6,158
Examined: Number	1,819	6,899	3,739	3,520
%	75.1%	69.9%	64.3%	57.2%
Mf positive: Number	790	18	2	0
%	43.43%	0.26%	0.05%	0.00%
95% confidence interval				
lower limit	41.14%	0.15%	0.01%	0.00%
upper limit	45.75%	0.41%	0.19%	0.10%
% of villages with:				
Prevalence <1%	0	95	90	100
Prevalence <5%	0	100	100	100

* Source: Diawara et al [17].

The entomological evaluations during phase 3A were done from September to December 2008, 16 to 19 months (about 1.5 years on average) after the last treatment in the main area and about 2.5 years on average after the last treatment in the test area. During phase 3B the entomological evaluations were done from August to December 2009, i.e. on average about 2.5 and 3.5 years after the last treatments in the main and test areas respectively. The results are given in table 2. More than 100,000 flies were collected and examined during phase 3A and phase 3B, but not a single infective fly was detected. The upper limit of the confidence interval for the infectivity rate remained for each catching point and for each evaluation year below the threshold of 0.5 F3H/1000 flies.

**Table 2:** Trend in vector infectivity rate in the R. Bakoye focus.

	Phase 1* (after 15 years Rx)	Phase 2* (1.5 years after last Rx in test area)	Phase 3A (1.5–2.5 years after last Rx)				Phase 3B (2.5–3.5 years after last Rx)			
Catching points	Infectivity rate (F3H/1000)	Infectivity rate (F3H/1000)	Flies examined	Infectivity rate (F3H/1000)	95% confidence interval		Flies examined	Infectivity rate (F3H/1000)	95% confidence interval	
Badala	**0.20**	**0.00**	7,200	**0.00**	0.00	0.26	7,500	**0.00**	0.00	0.26
Kolontan	**0.00**	**0.00**	16,500	**0.00**	0.00	0.12	21,300	**0.00**	0.00	0.09
Tieourou	**0.23**	**0.00**	10,500	**0.00**	0.00	0.18	10,500	**0.00**	0.00	0.18
Toufinko	**0.00**	**0.00**	15,600	**0.00**	0.00	0.12	17,400	**0.00**	0.00	0.11
Total	**0.14**	**0.00**	49,800	**0.00**	0.00	0.04	56,700	**0.00**	0.00	0.03

* Source: Diawara et al [17].

### R. Gambia focus, Senegal, 6-monthly treatment

In the R. Gambia focus, where treatment has been given at six monthly intervals, the last round of ivermectin treatment was given in May 2006 in the villages in the test area, and in all other villages in May 2007.

For phase 3, skin snip surveys had been planned for 40 villages. However, as reported previously, the population in the study areas was becoming increasingly reluctant to submit to the skin snip examination. This reluctance was particularly strong in the R. Gambia focus where the total population of 6 villages refused to participate in the final survey. Hence only 34 villages were surveyed, 18 in February 2009 during phase 3A, 21 months after the last treatment, and another 16 villages in May 2010 during phase 3B, 32 months after the last treatment in 10 villages and 48 months after the last treatment in 6 villages from the test area.

**Figure 6:**
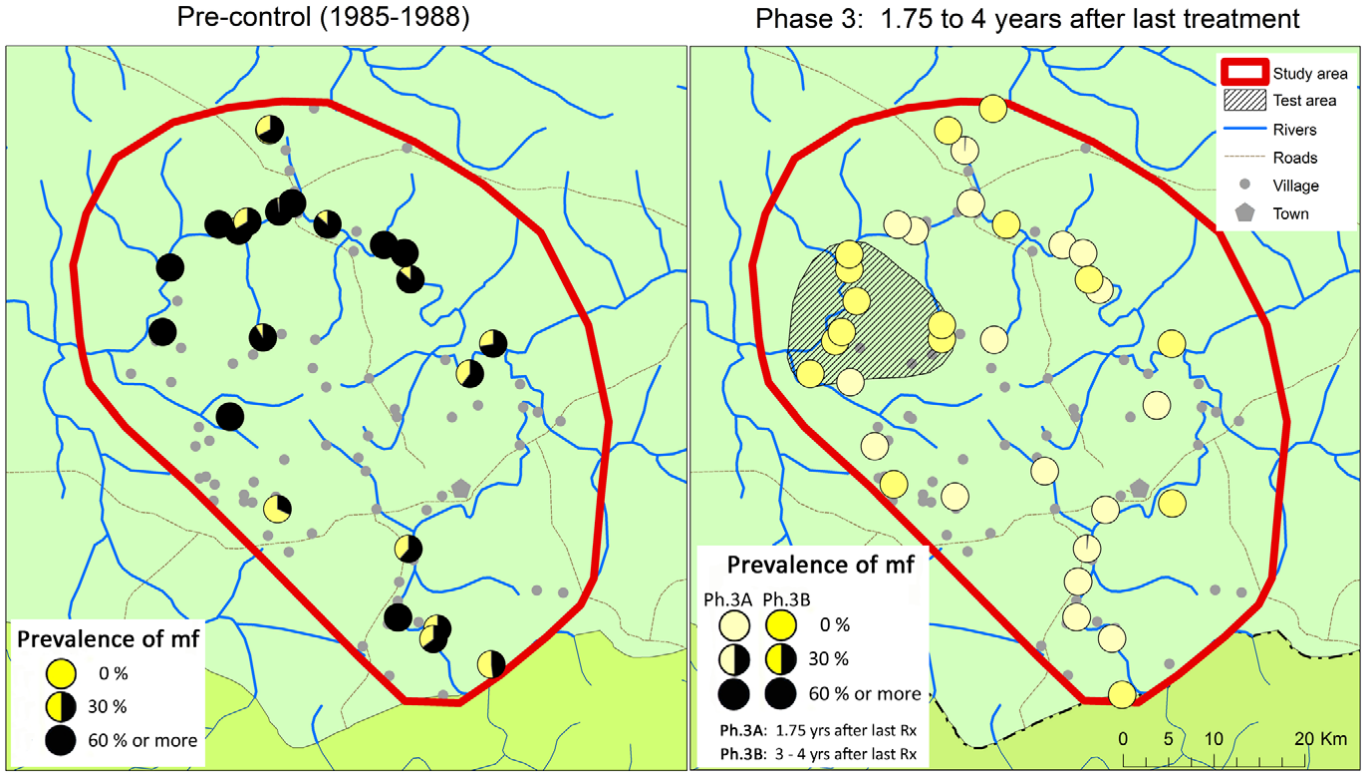
Prevalence of onchocerciasis infection in the R. Gambia focus before the start of treatment and 1.75 to 4 years after the last treatment round.

Figure 6 and table 3 show the results of the epidemiological surveys. During the treatment period the prevalence of mf had fallen from a precontrol prevalence of 49.5% to a prevalence of 0.06%, and the phase 3 results showed that the prevalence has remained at this very low level after the cessation of treatment. Only two mf positives were detected out of 1561 examined during phase 3A. Both were adult males (32 and 50 years of age) who had very low mf counts of 1 and 3 mf per skin snip. The 32 year old male reported to have been treated irregularly. The 50 year old male disappeared after the examination and could not be interviewed about his treatment history. During phase 3B there were no mf positives among the 1540 persons examined 32 to 48 months after the last treatment.

**Table 3:** Trend in prevalence of mf in the R. Gambia focus.

	Pre-control (1988–1990)*	Phase 1 (after 16 years Rx)*	Phase 3a (1.75 yrs after last Rx)	Phase 3b (3–4 yrs after last Rx)
Villages surveyed	23	42	18	16
Census population	3,487	7,184	2,655	3,445
Examined: Number	2,523	5,271	1,561	1,540
%	72.4%	73.4%	58.8%	44.7%
Mf positive: Number	1,250	3	2	0
%	49.54%	0.06%	0.13%	0.00%
95% confidence interval				
lower limit	47.58%	0.01%	0.02%	0.00%
upper limit	51.51%	0.17%	0.46%	0.24%
% of villages with:				
Prevalence <1%	0	95	90	100
Prevalence <5%	0	100	100	100

* Source: Diawara et al [17].

The entomological evaluations during phase 3A were undertaken from August to September 2008, about 1.5 years on average after the last treatment in the main area, and about 2.5 years on average after the last treatment in the test area. During phase 3B the entomological evaluations were undertaken exactly one year later, i.e. about 2.5 to 3.5 years after the last treatment in the main and test areas respectively. The results were again very clear: more than 150,000 flies were collected and examined during phase 3 and no infective fly was detected (table 4). The 95% confidence interval of the infectivity rates were for all catching points below the threshold of 0.05 F3H/1000 flies.

**Table 4:** Trend in vector infectivity rate in the R. Gambia focus.

	Phase 1* (after 17 years Rx)	Phase 2* (1.5 years after last Rx in test area)	Phase 3A (1.5–2.5 years after last Rx)				Phase 3B (2.5–3.5 years after last Rx)			
Catching points	Infectivity rate (F3H/1000)	Infectivity rate (F3H/1000)	Flies examined	Infectivity rate (F3H/1000)	95% confidence interval		Flies examined	Infectivity rate (F3H/1000)	95% confidence interval	
Bantacokouta	**0.06**	**0.00**	20,400	**0.00**	0.00	0.09	16,800	**0.00**	0.00	0.11
Sekoto	**0.00**	**0.00**	33,900	**0.00**	0.00	0.06	21,600	**0.00**	0.00	0.09
Soukouta	**0.00**	**0.00**	22,500	**0.00**	0.00	0.09	22,500	**0.00**	0.00	0.09
Yamoussa	**-**	**0.00**	6,900	**0.00**	0.00	0.28	12,300	**0.00**	0.00	0.16
Total	**0.02**	**0.00**	83,700	**0.00**	0.00	0.02	73,200	**0.00**	0.00	0.03

* Source: Diawara et al [17].

### R. Faleme focus, Mali/Senegal border, annual treatment

As mentioned in the methodology section above, the study design for the R. Faleme focus was modified because the phase 1 results for this focus did not fully meet the provisional criteria for stopping treatment. Phase 2 was therefore extended by one year during which two additional test areas were introduced in the south of the focus where treatment was stopped during phase 2B. When the follow-up results for these additional test areas showed no increase in infection and transmission levels during the first year after cessation of treatment, the focus was also moved into phase 3 and treatment was stopped in all villages. Hence, the cessation of treatment in the R. Faleme went in three steps: treatment was first stopped in the initial test area where the last treatment was given in May 2006, then in the additional two test areas where the last treatment was given in May 2007, and finally in all the remaining villages which received their last treatment in May 2008 in Mali and in October 2008 in the Senegal villages where the treatment was a few months delayed because of late arrival of ivermectin in the country during that year.

During phase 3 skin snip surveys were done in a total of 57 villages in the R. Faleme focus: 20 villages during phase 3A and 37 other villages during phase 3B. Because of the special epidemiologically situation in this focus, the number of survey villages for phase 3B was increased and all villages previously surveyed in phase 2 in the three test areas were included. During phase 3A, surveys were done in May 2010 in 20 villages that were all located outside the three test areas and which had their last treatment 19 to 24 months earlier. The 37 villages surveyed during phase 3B consisted of villages from all three groups: villages from the main area where the survey was done 31 to 36 months after the last treatment, villages from the additional test areas which were surveyed 48 months after the last treatment, and villages from the first test area which had not been treated for 60 months.

**Figure 7:**
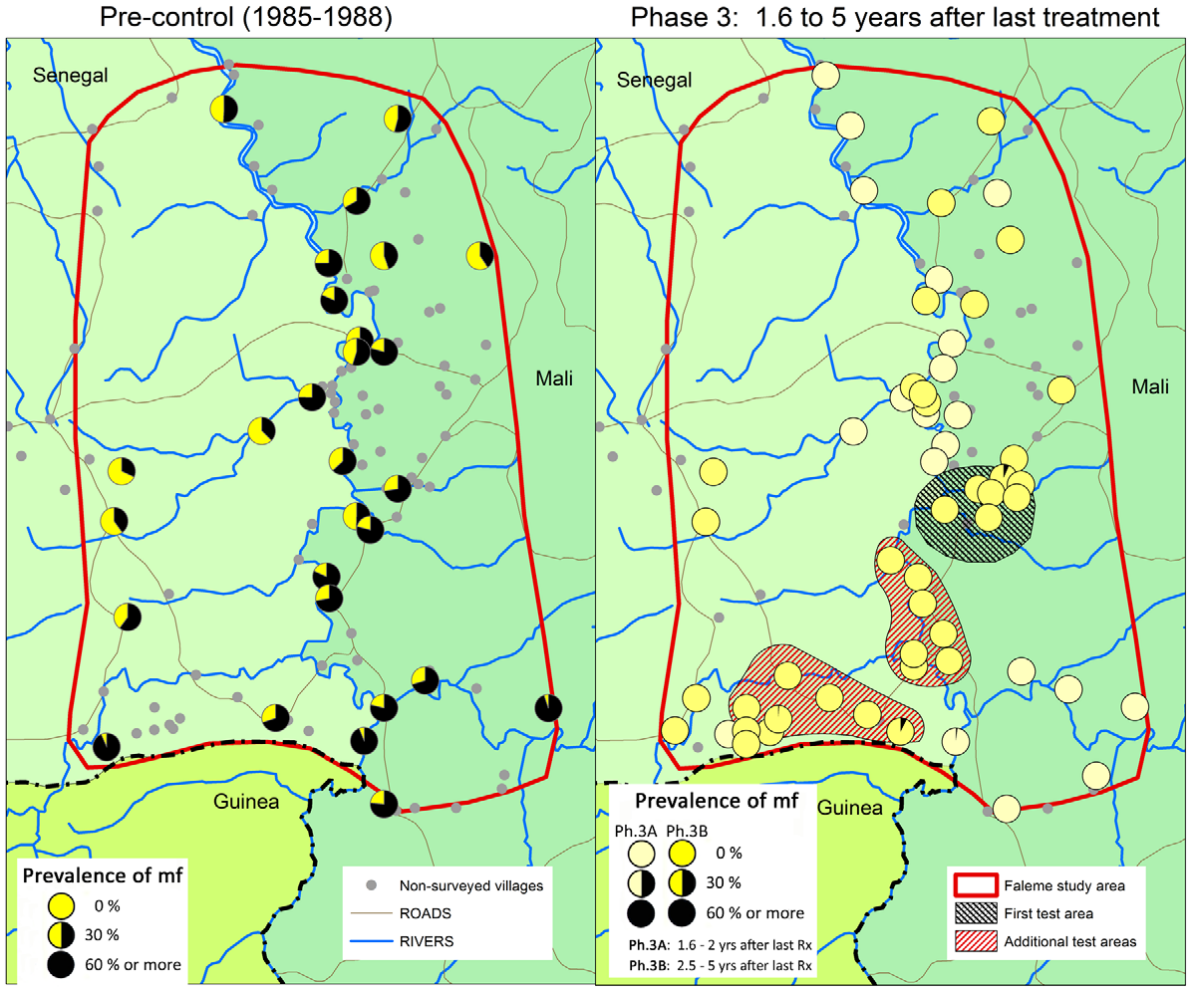
Prevalence of onchocerciasis infection in the R. Faleme focus before the start of treatment and 1.6 to 5 years after the last treatment round.

A summary of the epidemiological evaluation results is given in figure 7 and table 5. The prevalence of mf had fallen from a precontrol overall prevalence of 34% to a prevalence of 0.84% after 15 years of annual treatment. However, of the 44 villages surveyed during phase 1, 80% had a prevalence of mf <1% and 91% a prevalence of mf<5%. This did not meet the provisional thresholds for stopping treatment of at least 90% and 100% of villages in these two categories, and this was the reason for proceeding more prudently with stopping treatment in this focus and for the introduction of two additional test areas. When treatment was finally stopped, this was followed by a significant decline in the overall prevalence of mf to 0.13% in phase 3A, 1.5 to 2 years after the last treatment, and 0.07% in phase 3B, 2.5 to 5 years after the last treatment. The six persons who were mf positive (3 males and 3 females between the age of 19 and 49 years) had all low mf densities between 1.5 and 12 mf per skin snip. Four of them reported to have been treated irregularly with one having received only one treatment in 2006. The other two mf positives reported to have participated regularly in treatment. Five of the mf positives had been examined previously in phase 1 or phase 2, and four of those were already mf positive before the cessation of treatment. The fifth person, a 19 years old male, had been skin snip negative during two previous surveys but was now skin snip positive although with a very low mf density of 2 mf in the snip from the right iliac crest and 1 mf in the snip from the left iliac crest. This person was from a village in the south-west of the focus (figure 7) and had been treated irregularly. The distribution of the prevalence of mf by village showed that 95% of the villages had a prevalence <1% and 100% of villages a prevalence <5%, bringing the epidemiological evaluation results for the Faleme therefore also within the provisional threshold for elimination.

**Table 5:** Trend in prevalence of mf in the R. Faleme focus.

	Pre-control (1988–1990)*	Phase 1 (after 15 years Rx)*	Phase 3a (1.6–2 yrs after last Rx)	Phase 3b (2.5–5 yrs after last Rx)
**Villages surveyed**	28	44	20	37
**Census population**	5,567	8,106	3,438	8,241
**Examined: Number**	4,110	5,720	2,301	4,305
**%**	73.8%	70.6%	66.9%	52.2%
**Mf positive: Number**	1,411	48	3	3
**%**	34.33%	0.84%	0.13%	0.07%
95% confidence interval				
lower limit	32.88%	0.62%	0.03%	0.01%
upper limit	35.81%	1.11%	0.38%	0.20%
**% of villages with:**				
**Prevalence <1%**	0%	80%	95%	95%
**Prevalence <5%**	0%	91%	100%	100%

* Source: Diawara et al [17].

The entomological evaluations of phase 3A were undertaken from July to December 2009, on average about 1 year after the last treatment in the main area, 2.5 years after the last treatment in the additional test areas and 3.5 years after the last treatment in the first test area. During phase 3B the entomological evaluations were done one year later, and on average 2, 3.5 and 4.5 years after the last treatment in the different groups of villages.

The results of the entomological evaluations are summarised in table 6. This table also includes the entomological results for phase 2B after cessation of treatment in the two additional test areas.

**Table 6:** Trend in vector infectivity rate in the R. Faleme focus.

Catching points	Phase 1* (after 17 years Rx)	Phase 2A* (1 year after last Rx in 1^st^ test area)	Phase 2B (1.5 years after last Rx in additional test areas)	Phase 3A (1–3.5 years after last Rx)				Phase 3B (2–4.5 years after last Rx)			
Name	Infectivity rate (F3H/1000)	Infectivity rate (F3H/1000)	Infectivity rate (F3H/1000)	Flies examined	Infectivity rate (F3H/1000)	95% confidence interval		Flies examined	Infectivity rate (F3H/1000)	95% confidence interval	
Bambadji	-	0.00	0.00	18,900	0.00	0.00	0.10	17,400	0.06	0.00	0.30
Fadougou	0.00	0.12	0.00	21,900	0.00	0.00	0.09	19,200	0.00	0.00	0.10
Mahine Mine	0.11	0.00	0.00	24,900	0.00	0.00	0.08	16,500	0.06	0.00	0.31
Manankoto	0.00	0.00	0.00	20,700	0.00	0.00	0.09	15,900	0.00	0.00	0.12
Saroudia	0.23	0.00	0.00	13,800	0.00	0.00	0.14	20,400	0.00	0.00	0.09
Satadougou	0.00	0.19	0.00	21,900	0.00	0.00	0.09	17,700	0.00	0.00	0.11
Total	0.04	0.05	0.00	122,100	0.00	0.00	0.02	107,100	0.02	0.00	0.07

* Source: Diawara et al [17].

In each phase more than 100,000 blackflies were collected and examined, and no infective flies were found in phase 2B and phase 3A. In phase 3B there were two infective flies out of 107,100 flies examined, giving a very low infectivity rate of 0.02 F3H/1000 flies overall or 0.06 for each of the two positive catching points. The upper limit of the 95% confidence interval remains for all catching points below the threshold of 0.5 F3H/1000.

### Summary of study results

Table 7 gives a summary of the results for the main epidemiological and entomological indicators in the three study sites. In the R. Bakoye focus, the results are very clear. After the cessation of treatment at the end of phase 1, the infection and transmission levels continued to decline and 3 to 4 years after the last treatment both indicators were zero. For the R. Gambia focus, where treatment was given at six monthly intervals instead of annually, the results were equally clear and 3 to 4 years after the last treatment again no mf or infective larvae were detected. For the R. Faleme, where the prevalence of mf was significantly higher at the end of phase 1, there was also no sign of recrudescence after the cessation of treatment but instead there was a clear downward trend in the indicators and 3 to 5 years after the last treatment the prevalence of mf had fallen to extremely low levels, below 10% of the prevalence found in phase 1 before the cessation of treatment, while the vector infectivity rate had fallen below 5% of the provisional entomological threshold for elimination.

**Table 7:** Summary of main results.

Onchocerciasis Focus	Ivermectin Treatment	Indicator	Pre-control	Phase 1: 14–16 years treatment	Phase 3A: 1–3.5 years after last treatment	Phase 3B: 2–5 years after last treatment
R. Bakoye	Annual	Prevalence of mf (%)	43.4	0.26	0.05	0.00
		Vector infectivity rate (‰)	NA	0.14	0.00	0.00
R. Gambia	6-monthly	Prevalence of mf (%)	49.6	0.06	0.13	0.00
		Vector infectivity rate (‰)	NA	0.02	0.00	0.00
R. Faleme	Annual	Prevalence of mf (%)	34.3	0.84	0.13	0.07
		Vector infectivity rate (‰)	NA	0.09	0.00	0.02

NA: not available.

## Discussion

Annual or six monthly treatment with ivermectin of populations in onchocerciasis endemic areas has proven to be an effective strategy for controlling the disease as a public health problem. However, whether in the long term this strategy could also achieve elimination of onchocerciasis infection and transmission has not been clear. Until recently, most experts doubted that it would be feasible to achieve onchocerciasis elimination with ivermectin treatment in Africa where the vectors are very efficient and the disease is hyperendemic in many areas [13], [14], [16]. Although models predicted that elimination might be achieved in the long term, there was no empirical evidence to support this prediction and it was generally believed that elimination might not be possible in Africa [13], [14], [16].

The current study has fundamentally changed this perception. The first results of the study, as reported by Diawara et al [17], provided the first evidence that onchocerciasis elimination with ivermectin treatment is feasible in some foci in Africa. The current article reports the final results of the study and provides the definite evidence on the feasibility of elimination based on extensive data on onchocerciasis infection and transmission levels 2 to 3 years after stopping treatment in all villages in the three onchocerciasis foci, and 4 to 5 years after stopping treatment in parts of these foci.

The evaluation data show that after cessation of treatment there was no recrudescence of infection or transmission, but instead a continuously declining trend in infection and transmission levels up to 5 years after the last round of ivermectin treatment. In two sites, the prevalence of mf and the vector infectivity rate was zero during the final round of evaluation 3 to 4 years after the last treatment in these two areas. These results imply that the residual infection levels were so low that the vectors were no longer able to ingest and transmit the parasite, and that there was no renewed mf production by surviving worms up to 5 years after the last treatment. These results convincingly show that local elimination of onchocerciasis has been achieved in these two foci and that, as long as the parasite is not reintroduced, the population of this area will be forever free from the curse of onchocerciasis. Hence, the study has established the proof of principle that onchocerciasis elimination with ivermectin treatment is feasible in some endemic foci in Africa.

The results for the R. Faleme focus were even more remarkable. In this focus, the prevalence of mf was still relatively high at the end of the treatment period and did not completely meet the criteria for stopping treatment. However, these criteria were based on model predictions and experiences with stopping vector control, and they were still provisional criteria for ivermectin treatment to be tested in the current study. When the researchers discussed the phase 1 results for the R. Faleme focus with the Technical Consultative Committee of APOC, the committee recommended that the study should also proceed with stopping treatment and evaluating the subsequent trend in infection and transmission levels in the R. Faleme focus, given the provisional nature of the criteria and the operational importance of improved understanding of the feasibility of elimination and the risk of recrudescence in such borderline situations. It was therefore agreed to proceed with the study in the R. Faleme focus, but prudently through the introduction of two additional test areas that would be evaluated thoroughly for another year before stopping treatment throughout the focus. In view of this initial uncertainty, the final results for the R. Faleme focus were especially remarkable. After cessation of all treatment, the prevalence of mf continued to decline for a period of 3 to 5 years and the vector infectivity levels remained close to 0 throughout the follow-up period. The final results show that it was safe to stop treatment and that elimination has also been achieved in the R. Faleme focus.

It is important to note that the prevalence of mf was not equal to zero in any of the three foci when treatment was stopped, but that nevertheless there was no recrudescence of infection and transmission. This finding provides further evidence of the existence of breakpoints, as predicted by models, below which transmission cannot maintain itself and the infection will die out over time [31]. This is not the first time that empirical evidence of breakpoints has been produced. In virtually all areas where the OCP stopped vector control, the prevalence of mf was not yet zero and there were still several villages where the prevalence, although greatly reduced by vector control, was still in the range of 1 to 5% [32]. However, during the years after the cessation of vector control these residual infection levels declined to zero [33], [34]. Operationally these are important findings. It indicates that a few isolated infections, especially if it concerns only individuals with low mf counts, do not pose a significant threat and that treatment can be safely stopped as long as all indicators are below the elimination thresholds.

The study was undertaken by teams from the ministries of health of the two countries who used the regular epidemiological and entomological evaluation methods in which they had been trained by the OCP and that are the routine evaluation methods used in onchocerciasis control in Africa. In that sense, the study procedures were representative of those likely to be used by onchocerciasis control programs in other countries for future decision making on stopping ivermectin treatment. Nevertheless, our study was a carefully executed experiment with a stringent ethics protocol and one ethical requirement was to treat all individuals who were found to be mf positive during the skin snip surveys, even during surveys done after the cessation of treatment. It is unlikely that this requirement has had a significant impact on the study outcome as there were only very few mf positives detected after the cessation of treatment. In phase 3a, 7 mf positives were detected and treated (2 in R.Bakoye, 2 in R.Gambia and 3 in R.Faleme) but their treatment did not affect the results of any prevalence surveys because villages that were surveyed in phase 3A were not surveyed again in phase 3B. Furthermore, any possible impact on transmission would have been limited as phase 3A surveys were done in a sample of only 18% of villages in the study areas, and any mf positives in the other 82% of villages would have remained untreated during the study period. During phase 3B, 3 mf positives were detected but these were treated after the completion of the study and this did therefore not affect any study results.

It should be noted that one of the mf positives was an adult male from the R. Bakoye who had a relatively high microfilarial density of 58 mf per skin snip and who had been treated only twice. During phase 1, two persons with high microfilarial loads of 87 mf and 96 mf per snip were also detected in the R. Bakoye. It concerned two farmers who lived most of the year in hamlets on their farms on the river banks, near the Simulium breeding sites but very far from their village and they hardly ever received ivermectin. These findings underscores importance for CDTi programmes to ensure that ivermectin treatment reaches isolated high-risk groups such as fishermen and farmers living in hamlets near the river. One 19-year-old man from the R.Faleme, who had been skin snip negative during the surveys in phase 1 and phase 2, was diagnosed with a light infection of 1 and 2 mf in the skin snips from the left and right iliac crest respectively. He was reported to have been treated irregularly. This case might have been an isolated new infection resulting from low level transmission. Alternatively, it might represent a person with a very low intensity of infection at the border of detectability who was false negative during the surveys in 2006 and 2009. The latter explanation seems more plausible given that no other mf positives were detected in this village, nor in any of the surrounding 10 villages, and that the vector infectivity rate in the nearest catching point was zero throughout the study period. An important problem during the epidemiological surveys was the increasing reluctance of the population in the study villages to participate in the skin snip examination. This could have introduced a bias in the skin snip results if those who did not participate in the examination would also be more at risk of infection. Hence the importance of having also an extensive entomological evaluation as an independent measure of transmission levels in the study areas.

There is an ongoing debate about the potential value of a six monthly treatment strategy, as used by OEPA, instead of annual treatment strategy, as used in Africa, in order to reduce the total number of years treatment required to achieve elimination [35], [36]. The present study provides the only comparative data available to date on the long term impact of six monthly versus annual treatment. Our results show that both strategies achieved elimination after 15 to 17 years of treatment. Although the prevalence of mf in phase 1 was slightly lower in the R.Gambia focus, where treatment was given at six monthly intervals, than in the R. Bakoye focus and the R. Faleme focus where treatment has been annual, the final results after cessation of treatment were similar for all three sites, irrespective of treatment frequency. In each site there were still some mf positives after cessation of treatment, even in the R. Gambia focus after 34 treatment rounds, and transmission levels were zero or close to zero in all three sites. However, it is quite possible that elimination was achieved earlier in the R. Gambia focus but our study design does not allow us to determine exactly when the elimination threshold was reached.

One objective of the study was to develop and test a methodology and indicators for decision making on stopping ivermectin treatment. Our experiences indicate the importance of an approach that combines epidemiological and entomological evaluations as a basis for decision making to stop treatment and for subsequent evaluation to ensure that the decision to stop was correct. With respect to the epidemiological threshold for stopping treatment, the R. Faleme results seem to suggest that the current threshold is too conservative and that a higher threshold might be valid. However, onchocerciasis models predict that the risk of recrudescence depends on the precontrol endemicity level as an indicator of the local potential of transmission [10], [20], [37]. The precontrol endemicity levels in the R. Faleme were not very high, and for future evaluations we consider it prudent to maintain the current thresholds until sufficient empirical evidence has accumulated from multiple sites to justify their modification.

The results of the study have had significant impact on the strategy of APOC. Interim study results have been reported annually to the Technical Consultative Committee and Joint Action Forum of APOC. Based on the preliminary results, the Forum accepted that elimination may be feasible in at least some endemic areas, and requested APOC in December 2008 to generate the evidence to determine where and when treatment can be safely stopped [38]. APOC subsequently started an accelerated programme of systematic epidemiological evaluations of the long-term impact of ivermectin treatment and progress towards elimination in APOC projects that had at least 8 years of ivermectin treatment [39].

The current study was undertaken in hyperendemic onchocerciasis foci with seasonal transmission in the dry savanna of Mali and Senegal, and an important question is to what extent the results can be extrapolated to other endemic areas in Africa with different precontrol endemicity levels, transmission patterns and vector species. Computer simulations using the ONCHOSIM model indicate that the speed of decline in prevalence during the ivermectin treatment period, and thus the required duration of treatment to achieve elimination, depends greatly on the precontrol endemicity level. The results of the recent APOC evaluations of progress towards elimination have confirmed this [10], [40]. Hence it should not be concluded from the current study that 15–17 years are required everywhere to achieve elimination. In less endemic areas it should be possible achieve elimination in much shorter periods, maybe even less than 10 years, while it is predicted that in the areas with the highest endemicity levels up to 20 to 25 years of annual treatment may be required [10].

The main vector in the study areas is *S. sirbanum* which is the predominant vector of onchocerciasis in the dry savanna belt in West and Central Africa where transmission is limited to the rainy season [25], [41]. Hence, with respect to vector species and transmission patterns, our results appear representative for a vast area from Senegal to Sudan where millions of people were infected with *O.volvulus*. In the rest of Africa, the vectors include *S.damnosum* s.s. in the wet savanna, several other species of *S.damnosum s.l.* in forest areas where transmission is mostly perennial and *S.neavei* in parts of East and Central Africa [42]. The recent epidemiological evaluations by APOC have shown satisfactory progress towards elimination in the vast majority of ivermectin treatment projects in all these areas, and several projects with a population of over 7 million appear to have already reached the elimination breakpoint when treatment can be stopped [43], [44]. The results of these epidemiological evaluations are consistent with the results of phase 1 in our study. Nevertheless, due to differences in vector competence between vector species, the risk of recrudescence after cessation of treatment might still differ between endemic zones [45], [46]. We recommend, therefore, that the first ivermectin treatment projects that reach the elimination threshold in areas with different vector species or very high precontrol endemicity levels, proceed particularly carefully with stopping treatment using a methodology similar to that of our study, including detailed epidemiological and entomological evaluations of onchocerciasis infection and transmission levels for a period of 3 years after the cessation of treatment.

Recent years have seen a paradigm shift from onchocerciasis control to onchocerciasis elimination in Africa. The strategy of APOC has changed from control of onchocerciasis as a public health problem to a strategy of onchocerciasis elimination ‘where feasible’. A recent analysis has suggested that national elimination of onchocerciasis may be feasible in 23 African countries by the year 2020 [40], and a strategic plan to achieve this is under development [43]. The results of the current study have been instrumental for this evolution from onchocerciasis control to onchocerciasis elimination in Africa.
